# ENVIRONMENTAL EXPOSURES AND AGING OUTCOMES IN THE HRS USING CENSUS DATA

**DOI:** 10.1093/geroni/igae098.2075

**Published:** 2024-12-31

**Authors:** Joan Casey, Jenni Shearston, Taylor Mobley, L Paloma Rojas-Saunero, Juliet Zhou, Elizabeth Rose Mayeda

**Affiliations:** University of Washington, Seattle, Washington, United States; University of California Berkeley, Berkeley, California, United States; University of California Los Angeles, Los Angeles, California, United States; University of California Los Angeles, Los Angeles, California, United States; University of California Los Angeles, Los Angeles, California, United States; University of California Los Angeles, Los Angeles, California, United States

## Abstract

Incidence of Alzheimer’s disease and related dementias (ADRD) is 40–100% higher among Black compared with White Americans. Differential exposure to air pollution might partially explain this difference. We aim to determine if early-life exposure to poor air quality is related to late-life brain health among non-Hispanic Black and White Health and Retirement Study (HRS) participants. Using 1940 Census-linked enumeration district of residence, we estimate childhood exposure to fine particulate matter (PM2.5), traffic-related air pollution, and fossil fuel energy sources. We construct these exposures using archived climate model outputs from CMIP6 (PM2.5), gasoline sales, street networks, and oil and gas well and power plant locations paired with dispersion modeling techniques. Using multi-level survival analyses, we estimate the association between HRS participant childhood environmental exposures and late-life dementia. We also determine the contribution to Black-White disparities in dementia attributable to differences in early-life exposure. Preliminary analyses indicate that Black vs. White HRS participants had lower power plant emissions exposures but higher PM2.5 and traffic-related air pollution exposures in 1940. Regression analyses will evaluate whether 1940 environmental pollutant exposure is associated with dementia or dementia disparities by race. Identifying modifiable risk factors for ADRD could help protect cognitive health of the aging US population.

